# Effects of different doses of magnesium sulfate on pneumoperitoneum-related hemodynamic changes in patients undergoing gastrointestinal laparoscopy: a randomized, double-blind, controlled trial

**DOI:** 10.1186/s12871-019-0886-4

**Published:** 2019-12-20

**Authors:** Wei Tan, Dong-chen Qian, Meng-meng Zheng, Xuan Lu, Yuan Han, Dun-yi Qi

**Affiliations:** 1 Department of Anesthesiology, Yancheng Third people’s Hospital, Yancheng, China; 2 Department of Anesthesiology, The Affiliated Hospital of Xuzhou Medical University, Xuzhou, China; 3 Jiangsu Province Key Laboratory of Anesthesiology, Xuzhou Medical University, Xuzhou, China; 4 Xuzhou Medical College Affiliated Hospital, Xuzhou, 221002 China

**Keywords:** Magnesium sulfate, Systemic vascular resistance, Vasopressin, Pneumoperitoneum

## Abstract

**Background:**

The infusion of magnesium sulfate is well known to reduce arterial pressure and attenuate hemodynamic response to pneumoperitoneum. This study aimed to investigate whether different doses of magnesium sulfate can effectively attenuate the pneumoperitoneum-related hemodynamic changes and the release of vasopressin in patients undergoing laparoscopic gastrointestinal surgery.

**Methods:**

Sixty-nine patients undergoing laparoscopic partial gastrectomy were randomized into three groups: group L received magnesium sulfate 30 mg/kg loading dose and 15 mg/kg/h continuous maintenance infusion for 1 h; group H received magnesium sulfate 50 mg/kg followed by 30 mg/kg/h for 1 h; and group S (control group) received same volume 0.9% saline infusion, immediately before the induction of pneumoperitoneum. Systemic vascular resistance (SVR), cardiac output (CO), mean arterial pressure (MAP), heart rate (HR), central venous pressure (CVP), serum vasopressin and magnesium concentrations were measured. The extubation time, visual analogue scale were also assessed. The primary outcome is the difference in SVR between different groups. The secondary outcome is the differences of other indicators between groups, such as CO, MAP, HR, CVP, vasopressin and postoperative pain score.

**Results:**

Pneumoperitoneum instantly resulted in a significant reduction of cardiac output and an increase in mean arterial pressure, systemic vascular resistance, central venous pressure and heart rate in the control group (*P* <  0.01). The mean arterial pressure (T2 – T4)_,_ systemic vascular resistance (T2 – T3), central venous pressure(T3-T5) and the level of serum vasopressin were significantly lower (*P* <  0.05) and the cardiac output (T2 – T3) was significantly higher (*P* <  0.05) in group H than those in the control group. The mean arterial pressure (T4), systemic vascular resistance (T2), and central venous pressure(T3-T4) were significantly lower in group H than those in group L (*P* <  0.05). Furthermore, the visual analog scales at 5 min and 20 min, the level of vasopressin, and the dose of remifentanil were significantly decreased in group H compared to the control group and group L (*P* <  0.01).

**Conclusion:**

Magnesium sulfate could safely and effectively attenuate the pneumoperitoneum-related hemodynamic instability during gastrointestinal laparoscopy and improve postoperative pain at serum magnesium concentrations above 2 mmol/L.

**Trial registration:**

The study was retrospectively registered at Chinese Clinical Trial Registry; the registration number is ChiCTR-IPD-17011145, principal investigator: D.Y. Q., date of registration: April 13, 2017.

## Background

Although laparoscopic abdominal surgery has significant advantages, such as less trauma and faster recovery, the hemodynamic changes induced by pneumoperitoneum and the reverse Trendelenburg position are still challenges for anesthesia management during the surgery. The hemodynamic changes are characterized by abrupt elevations of arterial pressure and systemic vascular resistance. Besides the increase of intra-abdominal pressure, the increased levels of vasopressin, catecholamines, renin, and angiotensin are likely to be the reasons for these hemodynamic changes [1, 2]. These severe hemodynamic changes may have a significant impact on the perioperative status of the patient, especially in elderly patients with existing cardiovascular diseases. Therefore, it is crucial to use safe and effective drugs for maintaining hemodynamic stability during abdominal laparoscopy in such patients.

Magnesium sulfate is a well-known safe antihypertensive drug, which can be used during the perioperative period [3]. It can effectively attenuate the adverse hemodynamics fluctuations during laparoscopy, prevent the adverse cardiovascular events during laryngoscopy and tracheal intubation [4, 5], reduce the stress response, and strengthen the postoperative analgesia [6]. Furthermore, it was reported that high doses of intravenous magnesium sulfate could attenuate increased blood pressure and systemic vascular resistance [1, 4]. Although magnesium sulfate is believed to improve the cardiac output by reducing peripheral resistance, there is no available direct hemodynamic monitoring method to prove this effect.

We designed a double blinded, randomized, placebo-controlled clinical trial to investigate the possible association between the effects of magnesium sulfate and hemodynamic changes by using a FloTrac/Vigileo Monitoring System (Edwards Lifesciences, Irvine, CA, USA), which can monitor the cardiac output (CO), systemic vascular resistance (SVR), mean arterial pressure (MAP), central venous pressure (CVP) and heart rate (HR), and to determine the relationship between magnesium sulfate and vasopressin.

## Methods

### Participants and study design

We designed a double blinded, randomized, placebo-controlled clinical trial. The trial was in line with the CONSORT guidelines. The study was performed after receiving written informed consent from all participants. All study procedures were approved by the Clinical Research Ethics Committee of the Affiliated Hospital of Xuzhou Medical University, Jiangsu, China (the reference number: XYFY2017-KL005–01, approval date: December 18, 2017). The present trial was registered at http://www.chictr.org.cn (the registration number is ChiCTR-IPD-17011145, Principal investigator: D.Y. Q., date of registration: April 13, 2017).

### Patient management

Sixty-nine American Society of Anesthesiologists grade I and II patients, aged 30–65 years, undergoing laparoscopic partial gastrectomy with carbon dioxide pneumoperitoneum, were enrolled in this study. Patients with hypermagnesemia, with known allergy to magnesium sulfate, unstable blood pressure (hypertension or hypotension), cardiac dysfunction (NYHA grade III and IV), morbid obesity, and severe hepatic, renal or endocrine were excluded from the study. The Ethical Committee of the affiliated hospital of Xuzhou medical college approved the study (No: XYFY2017-KL005–01) and written informed consent was obtained from all participants.

Peripheral, central venous, and arterial cannulations were performed on the patients, under local anesthesia on arrival at the operation theatre. Electrocardiogram, oximetry, intra-arterial blood pressure, and central venous pressure were monitored. The participants were premedicated with midazolam, 1–2 mg intravenously, 10 min before the induction of anesthesia. The anesthesia was induced intravenously using etomidate 0.25 mg/kg and sufentanil 0.5 μg/kg. Endotracheal intubation was facilitated by administering the muscle relaxant cisatracurium 0.3 mg/kg intravenously. The initial tidal volume was 8–10 ml/kg at a respiratory rate of 12 breaths per minute. Ventilation was adjusted to maintain the end-tidal carbon dioxide at 35 to 45 mmHg. After 10 min of stable cardiovascular variables, HR, MAP, CO, CVP and SVR were measured using the FloTrac/Vigileo Monitoring System. The persons who dispensed the drugs and generated random sequence grouping did not participate in the monitoring of hemodynamic parameters and recruiting subjects. Immediately before the pneumoperitoneum, the participants were assigned (using a computer derived random number sequence) to one of the three groups. Group L received magnesium sulfate 30 mg/kg in 20 ml of normal saline over 5 min intravenously as a bolus dose followed by 15 mg/kg/h in 20 ml of normal saline as continuous maintenance infusion for 1 h; group H received magnesium sulfate 50 mg/kg in 20 ml of normal saline over 5 min as a bolus dose followed by 30 mg/kg/h in 20 ml of normal saline as continuous maintenance infusion for 1 h; and group S (control group) received 20 ml 0.9% saline infusion as bolus dose followed by 20 ml/h continuous maintenance infusion for 1 h, immediately before the induction of pneumoperitoneum.

Anesthesia in all the groups was maintained by propofol (4–6 mg/kg/h), remifentanil (0.25–0.35 μg/kg/min) and cisatracurium (0.1–0.12 mg/kg/h) administered intravenously. During the maintenance, bispectral index (BIS) values, determined by Conview™ Depth of Anesthesia Monitor (Pearlcare Medical, Zhejiang, China), were maintained at 45–60. During the surgery, we adjusted the pumping rate of propofol and remifentanil based on BIS, heart rate, and blood pressure, our study controlled according to the BIS value, When the BIS value was above 60 or below 45, propofol infusion rate would be adjusted by 0.5 mg/ kg/h each time. If the BIS value was maintained between 45 and 60, but the blood pressure fluctuates more than 20% of the basal level, remifentanil infusion rate would be adjusted by 0.02μg/kg/min each time. In addition, the degree of muscle relaxation was monitored with the TOF-GUARD muscle relaxometer (Organon Teknika, Turnhout, Belgium). Esophageal temperature was maintained using a heated blanket. Stopped pumping cisatracurium at the beginning of suture. The propofol and remifentanil infusions were stopped at the end of surgery. Patients were routinely sent to the PACU followed by intravenous administration of atropine sulfate 0.02 mg/kg and neostigmine 0.04 mg/kg for reversal of muscle relaxation, and the staffs worked in PACU monitored and removed the tracheal tube when the TOF ratio > 90%.

In cases of acute and severe hemodynamic fluctuations, the following medical interventions were performed: during the operation, we maintained the BIS value between 45 and 60 and excluded the effects of insufficient analgesia, for hypotension (MAP < 60 mmHg), an intravenous bolus dose of 50 μg phenylephrine was administered; and for hypertension (MAP > 110 mmHg) an intravenous bolus dose of 5 mg urapidil was administered. The data from the subjects who required vasoactive drugs during the surgery were excluded from the subsequent analysis.

#### The primary and secondary outcome

The primary outcome is the difference in SVR between different groups. The secondary outcome is the differences of other indicators between groups, such as CO, MAP, HR, CVP, vasopressin and postoperative pain score.

#### FloTrac/Vigileo monitoring system

The system is a minimally invasive continuous CO monitoring system based on arterial pressure waveform analysis. The catheter inserted into the radial artery was connected to the transducer of third-generation FloTrac/Vigileo system, which updated the MAP, HR, and CO every 20 s. The SVR can be continuously measured or calculated based on the patients’ information about age, gender, height, weight and the data on central venous pressure (CVP).

#### Surgical technique

The operations were performed in a standard group by a single surgeon. Carbon dioxide pneumoperitoneum was established in the patient in supine position, using a Verres needle. The pneumoperitoneal pressure was maintained at 15 mmHg throughout the laparoscopic procedure. According to the surgical requirements, all the patients were positioned in a head-up tilt of about 30°. To avoid potential severe hypotension as a result of anesthesia induction, all patients received 8–10 ml/kg compound electrolyte solution before the induction of anesthesia. Intraoperatively, the intravenous infusion of lactate ringer solution or compound electrolyte solution was 6 ml/kg/h. In order to fully observe the effect of magnesium sulfate on pneumoperitoneum, if the pneumoperitoneum duration in a participant was less than 2 h, then the data of the corresponding participant were removed from the final analysis.

#### Postoperative analgesia

In this trial, patients were not given regional analgesia and PCA analgesia pumps which are consisted mainly of sufentanil, dezocine and tropisetron were used after the patient left the PACU. We evaluated the VAS score 5 min and 20 min after extubation. When the score is > 5, we used fentanyl 0.05 mg iv for analgesia .

#### Evaluation of the outcome variables

The hemodynamic parameters of the three groups were recorded in the operation theatre using the FloTrac/Vigileo monitoring system. The recording of the hemodynamic data for each participant in each group was initiated after the induction of anesthesia and achievement of hemodynamic stability. The baseline values for all parameters were recorded at this point (T1). The study parameters measured included HR (beats/min), MAP (mmHg), CO (L/min), CVP (cmH_2_O) and SVR (dyn/s/cm^5^) at following intervals: 10 min after the induction of anesthesia in the supine position (T1); at the initiation of pneumoperitoneum (T2), and 5 (T3), 10 (T4), 30 (T5), and 60 (T6) minutes post-pneumoperitoneum in the reverse Trendelenburg position; 10 min after exsufflation in the supine position (T7). Blood samples for assessing serum magnesium and vasopressin concentrations were collected from the radial artery. The serum levels of vasopressin and magnesium were recorded at T1, T3, and T7. The dose of intraoperative remifentanil and fentanil, operation time, and pneumoperitoneum time were simultaneously recorded. Vasopressin levels were measured by radioimmunoassay (GC-911 Gamma radioimmunoassay counter, USTC ZONKIA, Anhui, china). We evaluated the extubation time (the time from the end of the operation to the extubation) and visual analogue scale (VAS) at 5 min and 20 min after extubation. The incidence of adverse reactions 24 h after the operation were also recorded.

### Statistical analysis

A sample size calculation was performed using PASS (Version 11.0; NCSS, USA) using a one-way analysis of variance. According to preliminary testing, we assumed that the mean SVR in Group S, Group L, Group H respectively are 2043, 1893, 1697, and the variability (SD) of the SVR of the three groups are 304, 297, 322. On the basis of a 0.05 level of significance with a power of 0.90, we sought to enroll at least 21 patients per group in the investigation to achieve sufficient statistical power. To compensate for the lack of 10% follow-up data, we aimed to recruit 23 patients per group.

Quantitative data confirming to the normal distribution were described as means ± standard deviation and the data of the non-normal distribution were represented by the median and the interquartile range. The data among the three study groups were analyzed by one-way analysis of variance (ANOVA) with post hoc least significant difference (LSD) test as appropriate. The Kruskal-Wallis test was used to analyze not normally distributed variables. For serially measured values, repeated-measures ANOVA and post hoc LSD tests were used to assess the trends in changes of serial values and interaction of trends between the groups. Comparison of continuous variables with baseline values were analyzed using student’s t-test in each group. Categorical variables were analyzed using chi-square (χ^2^) or and Fisher definite probability tests. *P* value < 0.05 was considered statistically significant. Statistical analysis was performed using statistical software SPSS16.0 (SPSS, Chicago, USA).

## Results

**Fig. 1:**
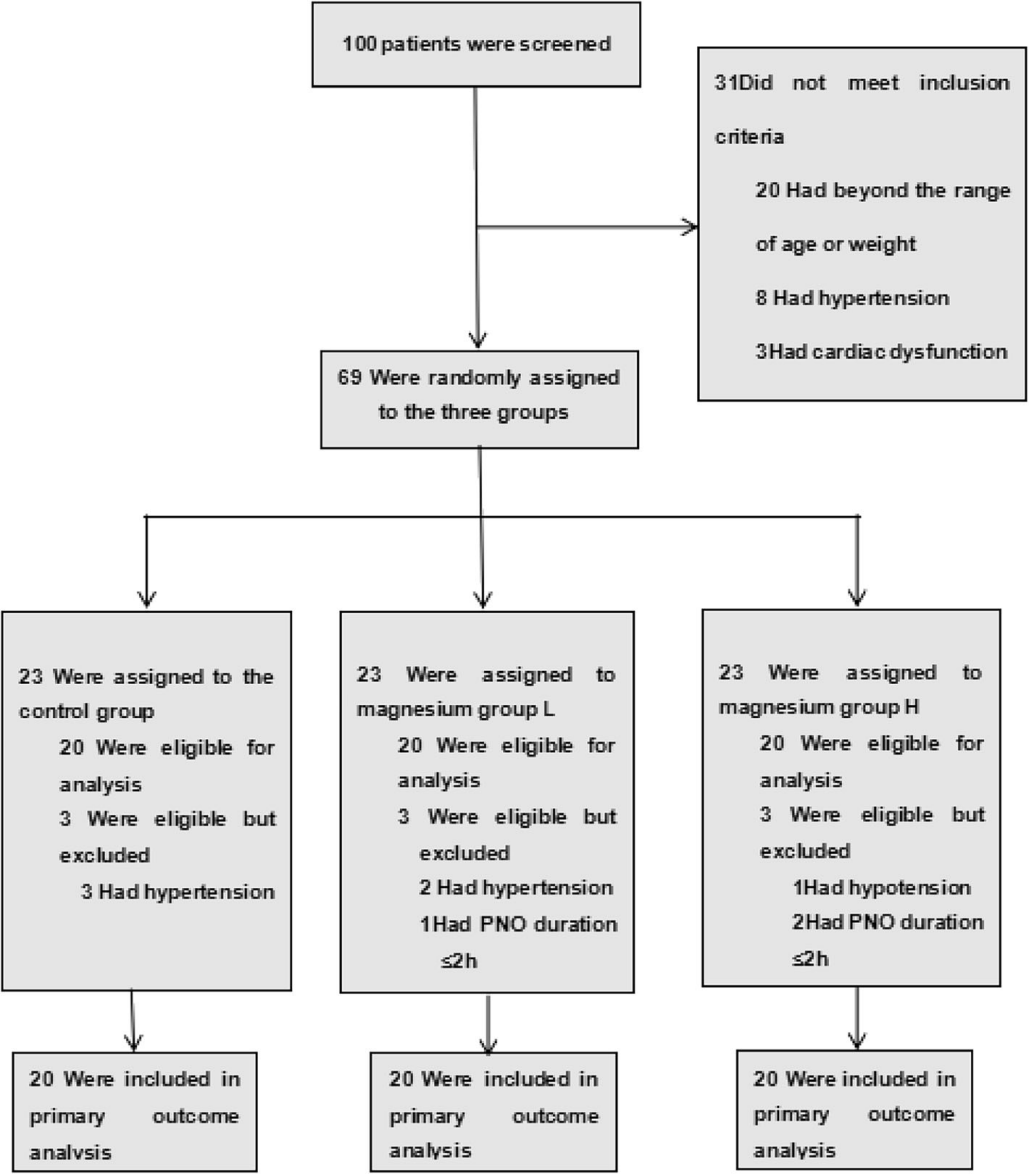
Flow chart for patient enrolment

The distribution of patients in the three study groups is shown in Table 1. All groups were comparable with respect to age, body weight, height, duration of surgery and pneumoperitoneum (h). The baseline MAP, HR, CO, SVR, CVP, vasopressin and preoperative medication were similar in all groups (Tables 1, 2). Three patients in the control group and two patients in group L required pharmacological management for hypertension. In addition, patients with pneumoperitoneum duration of < 2 h (one in magnesium group L and two in group H) were excluded. None of the patients in our study had bradycardia, while only one participant in group H had transient hypotension and improved after treatment with phenylephrine. Statistical analyses were performed with the remaining data (Fig. 1).

**Table 1:** Demographic characteristic of patients

	Group S	Group L	Group H	*P* value
Age (yrs)	58.6 ± 6.8	55.9 ± 7.2	56.2 ± 9.7	0.521
Height (cm)	170.0 (162.7, 172.0)	163.0 (160.5, 169.5)	170.0 (156.3, 173.0)	0.208
Weight (kg)	61.5 ± 6.6	59.8 ± 6	61.7 ± 7.4	0.603
operation time (h)	4.5 ± 0.4	4.4 ± 0.3	4.4 ± 0.3	0.789
PNO duration (h)	3.5 ± 0.3	3.4 ± 0.3	3.4 ± 0.2	0.298
HR (beats/min)	54.1 ± 5.7	54.2 ± 6.9	55.2 ± 6.2	0.822
MAP (mmHg)	77.9 ± 13.6	78.3 ± 11.2	79.8 ± 8.9	0.862
CO (L/min)	3.6 ± 0.5	3.7 ± 0.5	3.7 ± 0.5	0.706
SVR (dyn/s/cm^5^)	1571.8 ± 291.5	1563.5 ± 254.3	1582.2 ± 339.8	0.980
CVP (cmH_2_O)	7.8 ± 1.9	7.5 ± 1.4	7.6 ± 1.9	0.858
AVP (pg/ml)	199.6 ± 11.5	200.4 ± 14.5	200.2 ± 12.8	0.969
BIS	52.1 ± 4.3	54.5 ± 4.6	53.9 ± 4.5	0.199

HR, MAP, CO, SVR, CVP, BIS and AVP were measured at T1. *P*: differences between groups variation

**Table 2:** Preoperative medication in three group

	Group S (*n* = 20)	Group L (*n* = 20)	Group H (*n* = 20)	*P* value
Chronic beta-blockaden n (%)	3 (15%)	2 (10)	1 (5%)	0.625
ACE inhibitorsn n (%)	1 (5%)	1 (5)	2 (10%)	
calcium antagonistsn n (%)	0	1 (5%)	3 (15%)	

*P*: differences between groups variation

There was no significant difference in serum magnesium concentrations among the three groups at baseline. The average serum magnesium concentration level of group H was slightly higher than 2 mmol/l. Serum magnesium concentration level in group H rapidly increased to 2.01 ± 0.13 mmol/l (*P* <  0.01, compared with baseline value) at T3, then dropped to 1.38 ± 0.13 mmol/l at T7. In contrast, in group L, the level of serum magnesium concentration was 1.50 ± 0.11 mmol/l at T3, and it dropped to the same level as baseline at T7. Compared to group L, the serum magnesium concentration level was significantly (*P* <  0.01) higher at T3 and T7 in group H (Table 3).

**Table 3:** Serum magnesium concentrations (mmol/l) in three groups

	T1	T3	T7	*P*_*0*_ value
Group S	0.96 ± 0.06	0.93 ± 0.06	0.91 ± 0.05	< 0.001
Group L	0.96 ± 0.07	1.50 ± 0.11	1.07 ± 0.11	
Group H	0.98 ± 0.12	2.01 ± 0.13	1.38 ± 0.13	
*P*_*1*_	0.929	< 0.001	< 0.001	
95% CI	− 0.05~ 0.06	− 0.65~ − 0.51	− 0.23 ~ − 0.09	
*P*_*2*_	0.377	< 0.001	< 0.001	
95% CI	− 0.08~ 0.03	−1.15~ −1.02	− 0.54~ − 0.41	
*P*_*3*_	0.331	< 0.001	< 0.001	
95% CI	−0.08~ 0.03	− 0.57 ~ − 0.43	−0.37 ~ − 0.25	

Values were expressed as mean ± SD. *P*_0_:difference within group variation. *P*_*1*_: significance of difference between group S and group L; *P*_*2*_: significance of difference between group S and group H; *P*_*3*_: significance of difference between group L and group H

The changes in hemodynamic parameters are shown in Tables 4, 5, 6, 7 and 8.

**Table 4:** CO Changes During Laparoscopy

	T1	T2	T3	T4	T5	T6	T7	*P*_*0*_ value
Group S	3.6 ± 0.5	3.3 ± 0.5	3.3 ± 0.5	3.5 ± 0.8	3.5 ± 0.9	3.9 ± 0.9	4.8 ± 1.1	< 0.001
Group L	3.7 ± 0.5	3.4 ± 0.4	3.4 ± 0.4	3.6 ± 0.7	3.7 ± 0.7	4.0 ± 0.7	4.8 ± 0.9	
Group H	3.7 ± 0.5	3.6 ± 0.6	3.6 ± 0.6	3.6 ± 0.7	3.8 ± 0.9	4.0 ± 0.7	4.9 ± 0.9	
*P*_*1*_		0.501	0.400	0.549	0.464	0.921	0.934	
95% CI	−0.4~ 0.2	− 0.4 ~ 0.3	−0.4~ 0.2	− 0.6 ~ 0.3	−0.7 ~ 0.3	− 0.5~ 0.4	−0.5 ~ 0.6	
*P*_*2*_		0.045	0.049	0.673	0.231	0.752	0.777	
95% CI	−0.4~ 0.2	− 0.6 ~ 0.1	− 0.5~ 0.1	−0.5 ~ 0.4	− 0.8 ~ 0.2	−0.6 ~ 0.4	− 0.7 ~ 0.5	
*P*_*3*_		0.188	0.259	0.859	0.638	0.828	0.715	
95% CI	−0.3~ 0.3	− 0.5 ~ 0.1	− 0.5~ 0.2	− 0.4 ~ 0.5	−0.6 ~ 0.4	− 0.5~ 0.4	−0.7~ 0.5	

Values were expressed as mean ± SD. *P*_0_:difference within group variation. *P*_*1*_: significance of difference between group S and group L; *P*_*2*_: significance of difference between group S and group H; *P*_*3*_: significance of difference between group L and group H

**Table 5:** SVR Changes During Laparoscopy

	T1	T2	T3	T4	T5	T6	T7	*P*_*0*_ value
Group S	1571.8 ± 291.5	2042.8 ± 304.6	2113.2 ± 451.2	1933.0 ± 544.5	1783.0 ± 595.1	1592.1 ± 650	1361.4 ± 362.2	< 0.001
Group L	1563.5 ± 254.3	1893.6 ± 297.6	1955.4 ± 288.1	1816.8 ± 305.3	1622.1 ± 274.6	1590.4 ± 287.7	1358.9 ± 283.8	
Group H	1582.2 ± 339.8	1697.2 ± 321.9	1778.2 ± 291.5	1734.7 + 415.8	1631.2 ± 432	1534.6 ± 408.1	1303.2 ± 293.9	
*P*_*1*_		0.135	0.160	0.400	0.266	0.991	0.980	
95% CI	− 179.9 ~ 196.5	− 47.7~ 346.2	−65.2 ~ 380.5	− 158.1 ~ 390.3	− 126.1 ~ 447.8	− 297.9 ~ 301.4	− 197.1 ~ 202.1	
*P*_*2*_		0.010	0.040	0.153	0.294	0.702	0.562	
95% CI	− 198.7~ 177.7	148.6~ 542.6	112.1~ 557.8	− 76.0 ~ 472.4	−135.2 ~ 438.7	− 242.1 ~ 357.3	− 141.4 ~ 257.8	
*P*_*3*_		0.047	0.117	0.551	0.950	0.710	0.579	
95% CI	− 206.9~ 169.5	−0.64 ~ 393.4	−45.6~ 400.1	− 192.1~ 356.2	− 296.1 ~ 277.8	− 243.8 ~ 355.5	− 143.9~ 255.2	

Values were expressed as mean ± SD. *P*_0_:difference within group variation. *P*_*1*_: significance of difference between group S and group L; *P*_*2*_: significance of difference between group S and group H; *P*_*3*_: significance of difference between group L and group H

**Table 6:** MAP Changes During Laparoscopy

	T1	T2	T3	T4	T5	T6	T7	*P*_*0*_ value
Group S	77.9 ± 13.6	95.7 ± 12.9	95.2 ± 13.6	92.7 ± 10.3	84.9 ± 10.7	88.1 ± 9.7	86.6 ± 13	< 0.001
Group L	78.3 ± 11.2	92.2 ± 7.9	94.9 ± 8.6	91.0 ± 9.4	83.9 ± 9.8	86.9 ± 10.4	87.0 ± 12.6	
Group H	79.8 ± 8.9	87.5 ± 7.8	88.1 ± 7.9	84.4 ± 10.3	82.4 ± 10.7	82.7 ± 9.9	84.9 ± 12.7	
*P*_*1*_		0.265	0.927	0.592	0.773	0.706	0.912	
95% CI	−7.5~ 6.8	−2.7~ 9.7	− 6.3~ 6.8	−4.6~ 8.0	−5.6 ~ 7.5	− 5.1~ 7.5	− 8.6 ~ 7.6	
*P*_*2*_		0.010	0.048	0.011	0.450	0.096	0.685	
95% CI	−9.0 ~ 5.3	2.0~ 14.5	−0.3~ 12.7	2.0~ 14.6	−4.1~ 9.1	−0.9~ 11.6	−6.5 ~ 9.7	
*P*_*3*_		0.132	0.068	0.040	0.639	0.194	0.606	
95% CI	−8.7 ~ 5.7	− 1.5 ~ 10.9	−0.6~ 12.4	0.3 ~ 12.9	−5.0~ 8.1	−2.1 ~ 10.4	−6.0 ~ 10.2	

Values were expressed as mean ± SD. *P*_0_:difference within group variation. *P*_*1*_: significance of difference between group S and group L; *P*_*2*_: significance of difference between group S and group H; *P*_*3*_: significance of difference between group L and group H

**Table 7:** HR Changes During Laparoscopy

	T1	T2	T3	T4	T5	T6	T7	*P*_*0*_ value
Group S	54.1 ± 5.7	58.6 ± 10	57.7 ± 7.4	61.3 ± 7.1	62.9 ± 8.7	65.3 ± 10.2	61.3 ± 11.6	< 0.001
Group L	54.2 ± 6.9	56.1 ± 8.5	58.1 ± 8.1	60 ± 8.5	61.8 ± 9.3	65.1 ± 5.2	65.1 ± 5.2	
Group H	55.2 ± 6.2	57.2 ± 7.5	58.4 ± 9.1	57.8 ± 8.6	61.1 ± 8.7	61.4 ± 7.3	61.1 ± 7.7	
*P*_*1*_		0.350	0.879	0.614	0.671	0.920	0.798	
95% CI	−4.1 ~ 3.8	− 2.9 ~ 8.1	− 5.6 ~ 4.8	−3.8 ~ 6.4	− 4.4 ~ 6.8	− 4.7 ~ 5.2	− 6.5 ~ 5.0	
*P*_*2*_		0.601	0.775	0.171	0.513	0.116	0.945	
95% CI	−5.1 ~ 2.8	− 4.1~ 6.9	− 5.9~ 4.4	− 1.5~ 8.6	−3.7 ~ 7.4	− 1.0 ~ 8.9	− 5.6 ~ 6.0	
*P*_*3*_		0.679	0.894	0.384	0.818	0.141	0.745	
95% CI	−4.9 2.9	−6.6 4.3	−5.5~ 4.8	−2.8 ~ 7.3	− 4.9~ 6.2	−1.2 ~ 8.6	− 4.8~ 6.7	

Values were expressed as mean ± SD. *P*_0_:difference within group variation. *P*_*1*_: significance of difference between group S and group L; *P*_*2*_: significance of difference between group S and group H; *P*_*3*_: significance of difference between group L and group H

**Table 8:** CVP Changes During Laparoscopy

	T1	T2	T3	T4	T5	T6	T7	*P*_*0*_ value
Group S	7.8 ± 1.9	12.9 ± 1.7	10.4 ± 1.6	10.2 ± 1.4	10.2 ± 1.1	9.7 ± 1.0	7.4 ± 1.1	< 0.001
Group L	7.5 ± 1.4	12.7 ± 1.9	10.3 ± 1.4	10.1 ± 1.4	9.9 ± 0.9	9.4 ± 1.2	7.1 ± 1.0	
Group H	7.6 ± 1.9	12.4 ± 2.1	9.0 ± 2.3	8.9 ± 2.5	9.3 ± 2.1	9.5 ± 1.9	7.6 ± 1.4	
*P*_*1*_		0.804	0.792	0.801	0.514	0.436	0.500	
95% CI	−0.8 ~ 1.4	− 1.1 ~ 1.3	− 0.9 ~ 1.2	− 1.0~ 1.3	− 0.6 ~ 1.2	− 0.5~ 1.2	− 0.4~ 0.9	
*P*_*2*_		0.408	0.013	0.027	0.042	0.578	0.500	
95% CI	−0.9 ~ 1.3	− 0.7 ~ 1.7	0.3 ~ 2.5	0.2 ~ 2.5	0.1~ 1.8	−0.6~ 1.1	− 0.9~ 0.5	
*P*_*3*_		0.562	0.026	0.048	0.160	0.824	0.179	
95% CI	−1.2 ~ 1.0	−0.8~ 1.5	0.16 ~ 2.4	0.1~ 2.3	−0.2 ~ 1.5	− 0,9 ~ 0.7	−1.2~0.2	

Values were expressed as mean ± SD. *P*_0_:difference within group variation. *P*_*1*_: significance of difference between group S and group L; *P*_*2*_: significance of difference between group S and group H; *P*_*3*_: significance of difference between group L and group H

**Fig. 2:**
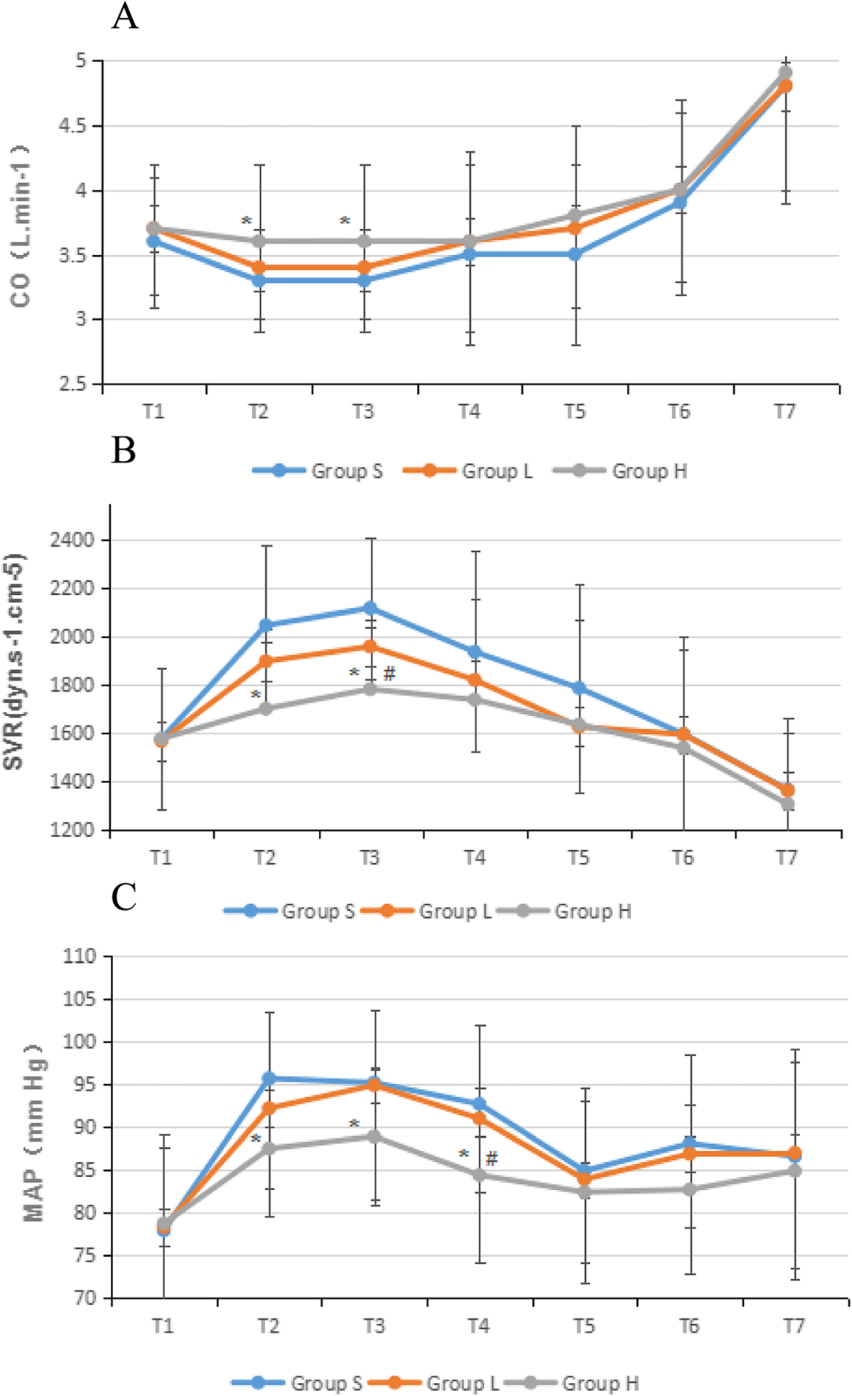
**a** Change in cardiac output (CO) during the laparoscopic gastrointestinal surgery. Values are expressed as mean (SD). *P < 0.05 when compared with the control group; #*P* < 0.05 when compared with group L. **b** Change in systemic vascular resistance (SVR) during the laparoscopic gastrointestinal surgery. Values are expressed as mean (SD). *P < 0.05 when compared with the control group; #*P* < 0.05 when compared with group L. **c** Change in mean arterial pressure (MAP) during the laparoscopic gastrointestinal surgery. Values are expressed as mean (SD). *P < 0.05 when compared with the control group; #*P* < 0.05 when compared with group L

Pneumoperitoneum instantly resulted in a significant reduction of CO and an increase in MAP, SVR, CVP, and HR in the control group (*P* <  0.01). Patients in group H showed stable levels of CO, SVR, CVP, and MAP. Compared to the control group, MAP (T2-T4), CVP(T3-T5) and SVR (T2-T3) were significantly lower in group H (*P* <  0.05), while the CO (T2-T3) was higher (*P* <  0.05). Compared to group L patients, MAP (T4), CVP(T3-T4) and SVR (T2) were significantly lower in group H (*P* <  0.05). There was no significant difference in HR between the three groups at each time point (Fig. 2).

The changes in plasma vasopressin concentrations are shown in Table 9.

**Table 9:** Vasopressin concentration Changes During Laparoscopy

	AVP (pg/ml)			BIS			TOF(%)		
	T1	T3	*P*_*0*_ value	T1	T3	*P*_*0*_ value	T1	T3	*P*_*0*_ value
								*P* value	
Group S	199.58 ± 11.50	230.68 ± 13.96	< 0.001	52.10 ± 4.30	52.20 ± 3.76	0.056	0	0	> 0.05
Group L	200.35 ± 14.47	216.84 ± 10.86		54.55 ± 4.60	53.00 ± 4.59		0	0	
Group H	199.91 ± 12.85	203.34 ± 17.20		53.95 ± 4.33	52.40 ± 4.75		0	0	
*P*_*1*_		0.003			0.567		*P* > 0.05		
95% CI	−9.0 ~ 7.46	4.7 ~ 22.8							
*P*_*2*_		< 0.001			0.886				
95% CI	−9.3 ~ 7.3	17.5 ~ 35.9							
*P*_*3*_		0.007			0.667				
95% CI	−8.5 ~ 8.1	3.7 ~ 22.1							

Values were expressed as mean ± SD. *P*_0_:difference within group variation. *P*: differences between groups variation. *P*_*1*_: significance of difference between group S and group L; *P*_*2*_: significance of difference between group S and group H; *P*_*3*_: significance of difference between group L and group H

**Fig. 3:**
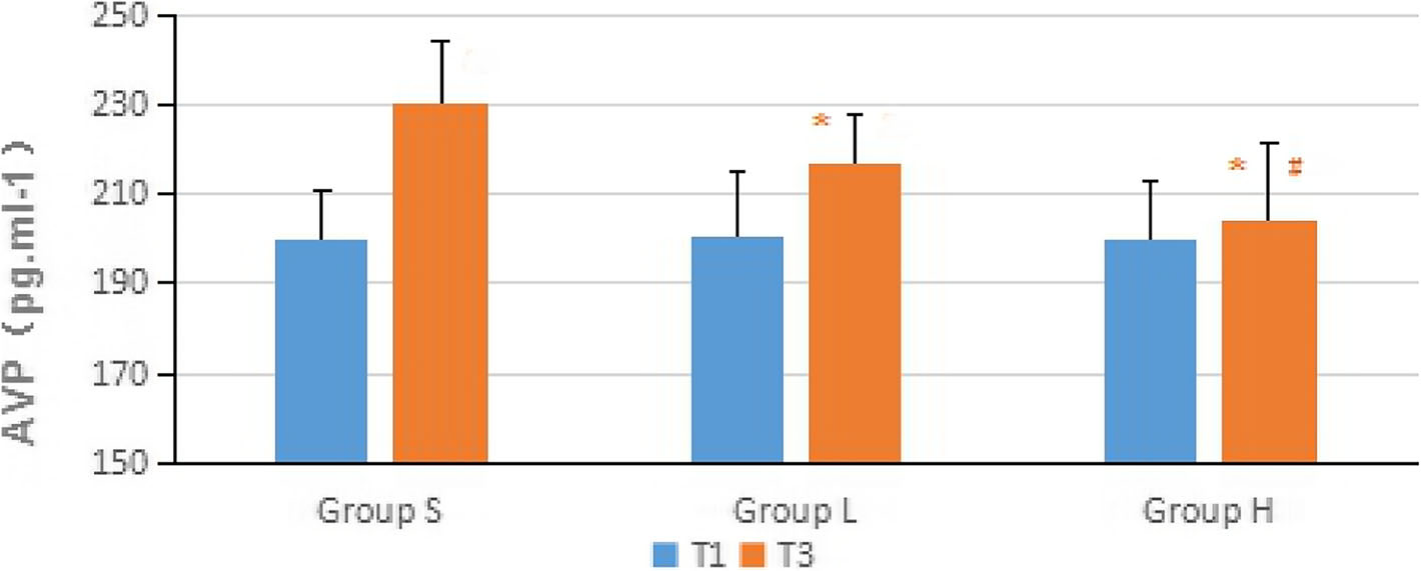
Changes in serum vasopressin during the laparoscopic gastrointestinal surgery. Values are expressed as mean (SD). *P<0.05 when compared with the control group; #*P* <0.05 when compared with group L

Compared with the baseline values, the level of vasopressin increased significantly in the control group and group L at T3 (*P* <  0.01). The level of vasopressin was significantly lower at T3 (*P* <  0.01) in group H compared to the control group and group L (Fig. 3).

The postoperative extubation time and the dosage of fentanil were not statistically significant between the groups. VAS (5 min), VAS (20 min), and the dosage of remifentanil were significantly decreased in group H compared to group L and the control group (*P* <  0.01). In addition, no postoperative muscle weakness and significant episodes of hypotension were found in any of the groups (Table 10).

**Table 10:** Perioperative and postoperative parameters among different groups

	Group S (*n* = 20)	Group L (*n* = 20)	Group H (*n* = 20)	*P* value
VAS _(5 min)_	4.65 ± 1.46	5.15 ± 1.45	3.15 ± 1.04	< 0.001 *P*_*1*_ 0.241 *P*_*2*_ 0.001 *P*_*3*_ < 0.001
VAS _(20 min)_	3.85 ± 0.93	4.30 ± 1.17	2.85 ± 0.88	< 0.001 *P*_*1*_ 0.161 *P*_*2*_ 0.003 *P*_*3*_ < 0.001
Remifentanil (ug/kg/min)	0.32 ± 0.02	0.32 ± 0.01	0.28 ± 0.01	< 0.001 *P*_*1*_ 0.656 *P*_*2*_ < 0.001 *P*_*3*_ < 0.001
Extubation time (min)	12.95 ± 4.22	11.75 ± 3.74	11.05 ± 4.75	0.368
Fentanil n(%)	5 (25%)	4 (20%)	1 (5%)	0.305

*P*: differences between groups variation *P*_*1*_: significance of difference between group S and group L; *P*_*2*_: significance of difference between group S and group H; *P*_*3*_: significance of difference between group L and group H

## Discussion

By using the FloTrac/Vigileo Monitoring System, the present study provides direct evidence regarding the attenuation of the changes in CO, SVR, CVP and MAP induced by pneumoperitoneum and maintenance of intraoperative hemodynamic stability by magnesium sulfate. Additionally, magnesium sulfate administered before pneumoperitoneum reduced the changes in vasopressin level associated with hemodynamic instability.

This is the first study to evaluate the direct effects of magnesium sulfate on CO and SVR using the FloTrac/Vigileo Monitoring System. Previous studies on the effects of magnesium sulfate on hemodynamic stability had used indirect or direct arterial pressure as a measure to indicate the peripheral circulatory resistance [1, 4, 5]. Compared to NIBP, both the thermodilution method and FloTrac / Vigileo can monitor SVR, but the thermometry measurement in clinical practice is limited, mainly because of the risks associated with pulmonary artery catheterization and it can not provide continuously monitor changes in SVR. In contrast, the FloTrac / Vigileo is a minimally invasive hemodynamic monitoring system, the hemodynamic parameters such as CVP, MAP, SVR and CO can be continuously measured.

The pneumoperitoneum lifts the diaphragm, causing an increase in intrathoracic pressure and venous resistance, thereby reducing CO. The body sympathetic excitement compensatory raises SVR to maintain arterial blood pressure. However, the increase in SVR further decreases the CO, forming a vicious circle [7, 8]. Younger patients can tolerate the decrease in CO under physiologic conditions. As the blood vessels progressively harden and cardiovascular compensatory function declines, their ability to adapt to changes in circulating blood volume is severely reduced in elderly. For example, a further decrease in CO induced by pneumoperitoneum might result in deleterious effects in elderly patients with hypertension or ischemic heart disease.

By using the FloTrac / Vigileo Monitoring System, our study demonstrated that pneumoperitoneum decreased CO, which was observed in previous studies [7]. Our results also showed that intravenous magnesium sulfate at a dose of 50 mg/kg could effectively alleviate the reduction in CO, by dilating the peripheral blood vessels and reducing the vascular tone.

During pneumoperitoneum for operative laparoscopy, impairment of hemodynamic status occurs mainly at the beginning of peritoneal insufflation [2]. It is well known that elevated intrapleural pressure significantly reduces the venous return and the circulating blood volume, which induces the elevated levels of vasopressin [1, 2, 9, 10]. Adrenergic receptor blockers, calcium channel blockers, opioids, and vasodilators are routinely used to attenuate the pneumoperitoneum-related hemodynamic instability, but they are all accompanied with varying degrees of reduction in CO. In contrast, magnesium sulfate produces rapid and transient vasodilation by a direct action without causing a reduction in CO, and by indirectly blocking the sympathetic pathway and inhibiting the catecholamine and vasopressin release [1, 3]. Adjuvant analgesia with magnesium sulfate can significantly reduce the dose of remifentanil. Consistent with these findings, in the present study, at the initiation of pneumoperitoneum, and 5, 10 min post-pneumoperitoneum are the most severe periods of hemodynamic fluctuations, and it was also the most effective time period for magnesium sulfate to inhibit pneumoperitoneum associated hypertension. Moreover, hemodynamic fluctuations at 30 and 60 min post-pneumoperitoneum were less pronounced, indicating that magnesium sulfate only reduced abnormally elevated blood pressure and had no effect on normal blood pressure. Jee D found that intravenous magnesium sulfate could improve the increased arterial pressure and inhibit the release of vasopressin caused by pneumoperitoneum at 5 and 10 min post-pneumoperitoneum [1]. Similarly, we found that magnesium sulfate at a dose of 50 mg/kg could effectively attenuate the release of vasopressin.

A minimum therapeutic level of 2 mmol/L magnesium sulfate has been proposed in the clinical management of eclampsia patients [11]. If the magnesium serum concentration is more than 3 mmol/L, the patients may develop tendinous reflexes [3]. Therefore, it is essential to select a safe and effective minimum dose of magnesium sulfate to ensure the safety of patients. Besides, taking into account the effect of magnesium sulfate on intraoperative muscle relaxation, the magnesium ion concentration was measured again before extubation to ensure patients’ safety. In the present study, the average serum concentration of magnesium sulfate in group H was between 2 and 3 mmol/L. In group L, the level of serum magnesium concentration was lower than 2 mmol/L at T3. Further, there were no statistically significant differences in the extubation time between the three groups, did not observed any reported serious adverse effects and the potentiation effect of magnesium on neuromuscular blockade as reported in other observations [12], which could be related to the surgery time and the metabolic duration of magnesium sulfate. These results indicate that 50 mg/kg magnesium sulfate may be a safe dose for attenuating the pneumoperitoneum-related hemodynamic changes during laparoscopic gastrointestinal surgery.

The analgesic effect of magnesium sulfate at a dose of 50 mg/kg was relatively obvious, which may be related to the higher concentration of magnesium ions after surgery (Serum magnesium concentration level in group H was1.38 ± 0.13 mmol/l after surgery). In contrast, the reason why the postoperative pain score in group L was higher was that the magnesium ion concentration was lower after surgery, so it did not play an analgesic role. (The level of serum magnesium concentration was 1.07 ± 0.11 mmol/l after surgery). Perioperative intravenous magnesium reduced opioid consumption and pain scores, which was believed to be caused by a physiological block of the ion channel on the N-methyl-D-aspartate receptor and inhibition of the intracellular Ca^2+^ mobility [6, 10, 13]. This analgesic effect may also contribute to the hemodynamic stability in the patient during surgery. However, further research is needed to determine the exact mechanisms causing the analgesia.

## Conclusion

Magnesium sulfate is a safe, inexpensive, and old drug for treating hypertension. In recent years, it has been found to have many other effects during perioperative applications, such as its role in alleviating post-operative pain and treating intubation induced hypertension. In the present study, by comparing different dose of magnesium sulfate, we found that the application of 50 mg/kg magnesium sulfate not only can suppress stress response and hypertension significantly caused by laparoscopic surgery, but also has analgesia effect after surgery. Another advantage of this trial is that, we firstly provided the direct evidence for the role of magnesium sulfate in suppressing the increased SVR induced by pneumoperitoneum. There are several limitations of this study. First, it is a single-center study. Second, we did not monitor the release of catecholamines during the surgery. Other studies have showed that perioperative administration of magnesium sulfate could reduce the release of catecholamines induced by intubation [4].

## Data Availability

The datasets generated during the current study are not publicly available due the regulation of data management of Xuzhou Medical College Affiliated Hospital, but are available from the corresponding author on reasonable request.

## Abbreviations

ANOVA: Analysis of variance
BIS: Bispectral index
CO: Cardiac output
CVP: Central venous pressure
HR: Heart rate
MAP: Mean arterial pressure
SVR: Systemic vascular resistance
VAS: Visual Analogue scale
